# Ecological momentary insights into cannabis use and its contexts: a pre-post legalization study in Germany

**DOI:** 10.1038/s41598-026-70148-7

**Published:** 2026-09-05

**Authors:** Louisa Diallo, Gianna Spitta, Samanda Krasniqi, Nadja Samia Bahr, Sabrina Doerr, Dominic Reichert, Markus Reichert, Jan-Luca Wiedenhoeft, Ulrich Ebner Priemer, Tobias Banaschewski, Andreas Heinz, Fabian Arntz, Michael A. Rapp

**Affiliations:** 1 Department of Psychiatry and Neurosciences, Charité Campus Mitte, Charité - Universitätsmedizin Berlin, Charitéplatz 1, Berlin, 10117 Germany; 2 German Center for Mental Health (DZPG), Berlin, Germany; 3 Department of Psychiatry and Psychotherapy, Central Institute of Mental Health, Medical Faculty Mannheim/Heidelberg University, Mannheim, Germany; 4 Department of Sport and Exercise Science, Faculty of Natural and Life Sciences, University Salzburg, Salzburg, Austria; 5 Department of Social and Preventive Medicine, University of Potsdam, Potsdam, Germany; 6 Institute of Sports and Sports Science, Karlsruhe Institute of Technology, Karlsruhe, Germany; 7 Child and Adolescent Psychiatry and Psychotherapy, Central Institute of Mental Health, Medical Faculty Mannheim, University of Heidelberg, Mannheim, Germany; 8 Department of Psychiatry and Psychotherapy, Technical University of Dresden, Dresden, Germany; 9 Department of Psychiatry and Psychotherapy, University of Tübingen, Tübingen, Germany; 10 Department of Psychiatry and Psychotherapy, Central Institute of Mental Health, Mannheim, Germany

**Keywords:** Cannabis use, Cannabis legalization, Ecological momentary assessment, Germany, Environmental sciences, Health care, Psychology, Psychology

## Abstract

Globally, cannabis ranks third in psychoactive substance prevalence after nicotine and alcohol, with heavy use linked to psychosis, anxiety, and depression. However, real-time data on how legal reforms influence consumption remain scarce. This Ecological Momentary Assessment (EMA) study analyzed daily cannabis use patterns before and after Germany’s recreational cannabis decriminalization in April 2024 in 49 individuals (34.7% women, age 33.8 ± 11 years). Following the legal reform, a selective increase in consumed amounts among younger individuals (β = 0.01, 95%CI = 0.00–0.02, z = 3.05) was observed. Further, consumed amounts increased in outdoor (β = 0.19, 95%CI = 0.04–0.34, z = 2.51) but decreased in public settings (β = -0.46, 95%CI = -0.66 - -0.25, z = -4.36), and consumption was more likely to occur on holidays (β = 3.91, 95%CI = 1.68–9.08, z = 3.17). All ps < 0.05. By capturing the immediate post-legalization period, this study contributes to important early insights to inform prevention, treatment, and regulation.

**Supplementary Information:**

The online version contains supplementary material available at https://doi.org/10.1038/s41598-026-70148-7.

## Introduction

Cannabis ranks third among psychoactive substances in global prevalence after tobacco and alcohol^1^. Over the past three decades in Germany, cannabis use and misuse have steadily increased^2^. This increase is clinically relevant given the dose-dependent harms associated with cannabis use which include elevated risks for psychosis, anxiety, and depression, alongside physical health consequences^3–6^. However, these potential mental and physical health risks must be considered within the broader context of harms associated with the criminalization and decriminalization of cannabis^7^.

Whether legalization or criminalization more effectively reduces cannabis-related harms remains debated, but many high-income countries are shifting toward legalization^8^. Recreational cannabis use is legal in Canada, Uruguay, Malta, and many U.S. states, while various forms of decriminalization exist in countries like the Netherlands, Portugal, and parts of Australia^9^. As of April 2024, Germany implemented legislation allowing adults (aged 18 and older) in Germany to possess up to 25 g (g) of cannabis in public, 50 g at home and cultivate up to three cannabis plants. Since January 2025, certain previous convictions related to cannabis offenses, that are no longer illegal, have been removed from criminal records^10,11^. To our knowledge, no real-time data on the impact of cannabis legalization in Germany exist. Furthermore, high-resolution data on cannabis use more broadly remain rare. We aimed to address this gap by investigating daily consumption patterns among non-medical users using Ecological Momentary Assessment (EMA), which captures cannabis use in real time and natural settings via smartphone, enhancing ecological validity. It is well documented that real-time data provide valuable insights into consumption behavior and contextual factors, offering advantages over retrospective assessments^12,13^. Drawing on recent findings, we hypothesized an overall increase in cannabis use following the legal reform^14–16^. In particular we expected a shift toward outdoor and public settings, due to a reduction in perceived criminalization^17^. To better understand how legalization might influence consumption patterns, we also examined whether cannabis use began to resemble patterns typically observed for alcohol. Prior EMA studies, including findings from our own research group, have demonstrated robust temporal patterns in alcohol use, with marked increases on weekends and public holidays (e.g., Christmas or New Year’s Eve), and consistent reductions in January, often referred to as the “Dry January effect”^18,19^. Derived from this, we investigated whether similar temporal dynamics would emerge in cannabis use following legalization. Given that younger individuals are considered particularly vulnerable to cannabis-related mental health risks^20–22^, we also explored age-related differences. Up to now only a limited number of EMA studies have explored contextual factors in cannabis use, showing that consumption tends to increase in social settings, while location played a comparatively minor role^23,24^. For alcohol, both laboratory and EMA studies indicate higher consumption in social compared to solitary contexts^25–27^. Building on these findings, we expected stable baseline patterns of higher consumption in group settings, along with legalization-related increases in social use.

This study provides real-world insights into daily cannabis use and context-specific changes following legalization, helping to address a critical research gap and offering evidence to inform clinical care and future research.

## Results

In total, 452 participants were contacted, and 78 participants were screened, of which 61 participants were enrolled in the study (38 men, 21 women, 2 participants identifying as non-binry) ranging in age from 20 to 60 years (*mean* = 32.9, *SD* = 10.3) (see Supplement Figure S1).

After exclusion of the participants that did not participate post-legalization or did not provide sufficient data, the final sample consisted of 49 participants (31 men, 17 women, 1 non-binary), age range 20–60 years (*mean* = 33.8, *SD* = 11). Sample characteristics as well as number of observations, consumption days and consumption amount pre- and post- legalization are reported in Table 1.

**Table 1:** Characteristics of the included participants.

Number of participants	49	
Gender	17 women | 31 men | 1 non-binary	
Age [years]	33.8 ± 11	
	Pre-legalization	Post-legalization
Baseline assessments		
AUD criteria [sum-score]	1.2 ± 1.9	0.4 ± 0.8
AUDIT [sum-score]	5.1 ± 2.5	4.8 ± 2.4
CUDIT [sum-score]	9.9 ± 7	9.3 ± 7
Ecological momentary assessments		
Number of observations	1535	1485
Cannabis consumption days (in %)	696 (45.3)	685 (46.1)
Consumed amount per consumption day (g)	0.43 ± 0.42	0.52 ± 0.46

AUD = alcohol use disorder criteria (DSM 5); AUDIT = Alcohol Use Disorder Identification Test^40^; CUDIT = Cannabis Use Disorder Identification Test^41^; Pre-post differences in AUD, AUDIT & CUDIT were estimated using a nested linear regression design; Values are presented as means ± standard deviation.

### Attrition and compliance

During the pre-legalization assessment, 61 participants provided daily information on cannabis consumption. 85.2% completed at least 90% of the assessment period (i.e., 14.8% attrition), and overall compliance to daily EMA prompts was 80 ± 20.3%. Post-legalization, 49 participants provided daily cannabis consumption information, of which 79.6% completed at least 90% of the planned 6-week assessment period (i.e., 20.4% attrition). Compliance rates in this phase were 84.3 ± 20.4%.

### Cannabis use behavior and consumption patterns

**Fig. 1:**
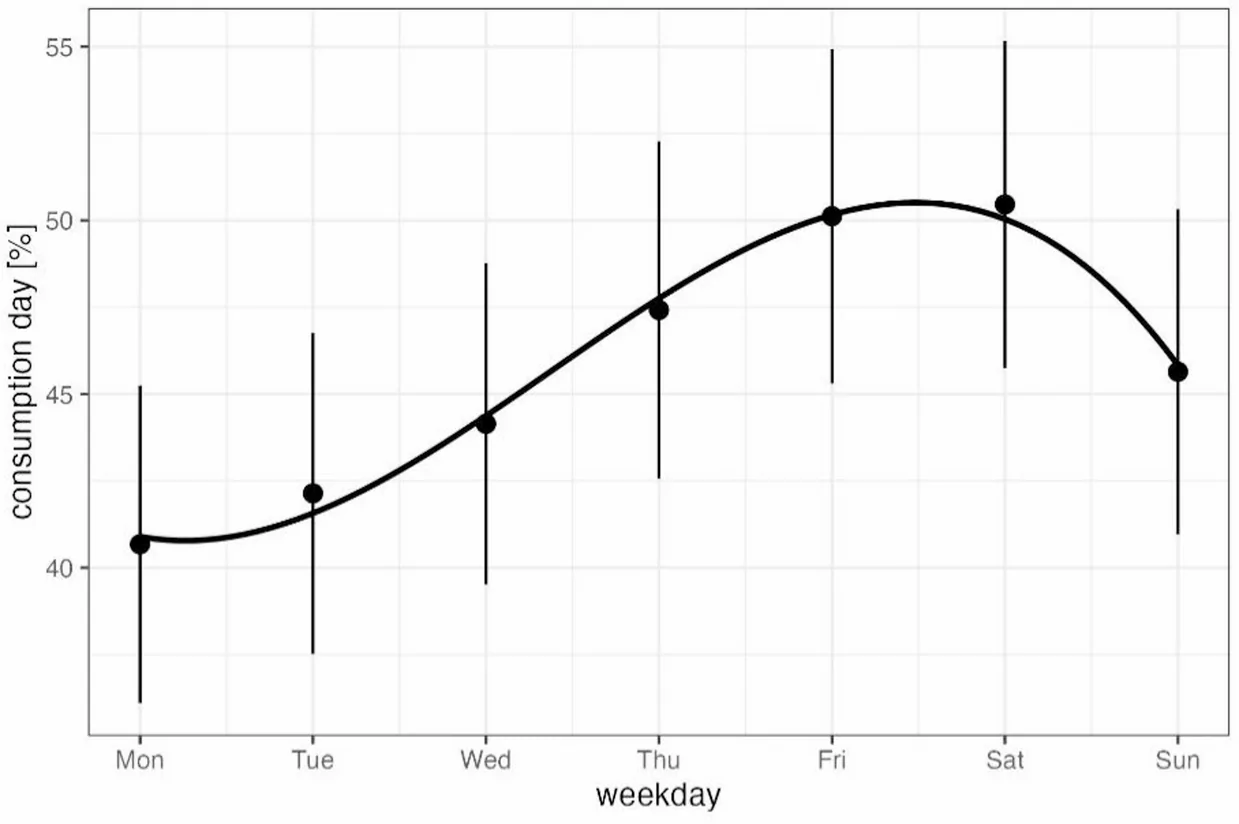
Cannabis consumption patterns across the week. Cannabis consumption frequency at baseline (pre-legalization) is shown in % across each day of the week showing significantly higher likelihood for consumption on weekends (days preceding a weekend day) than weekdays (days preceding a week day) (β = 1.54, 95%CI = 1.12–2.12, z = 2.66, model 1).

**Fig. 2:**
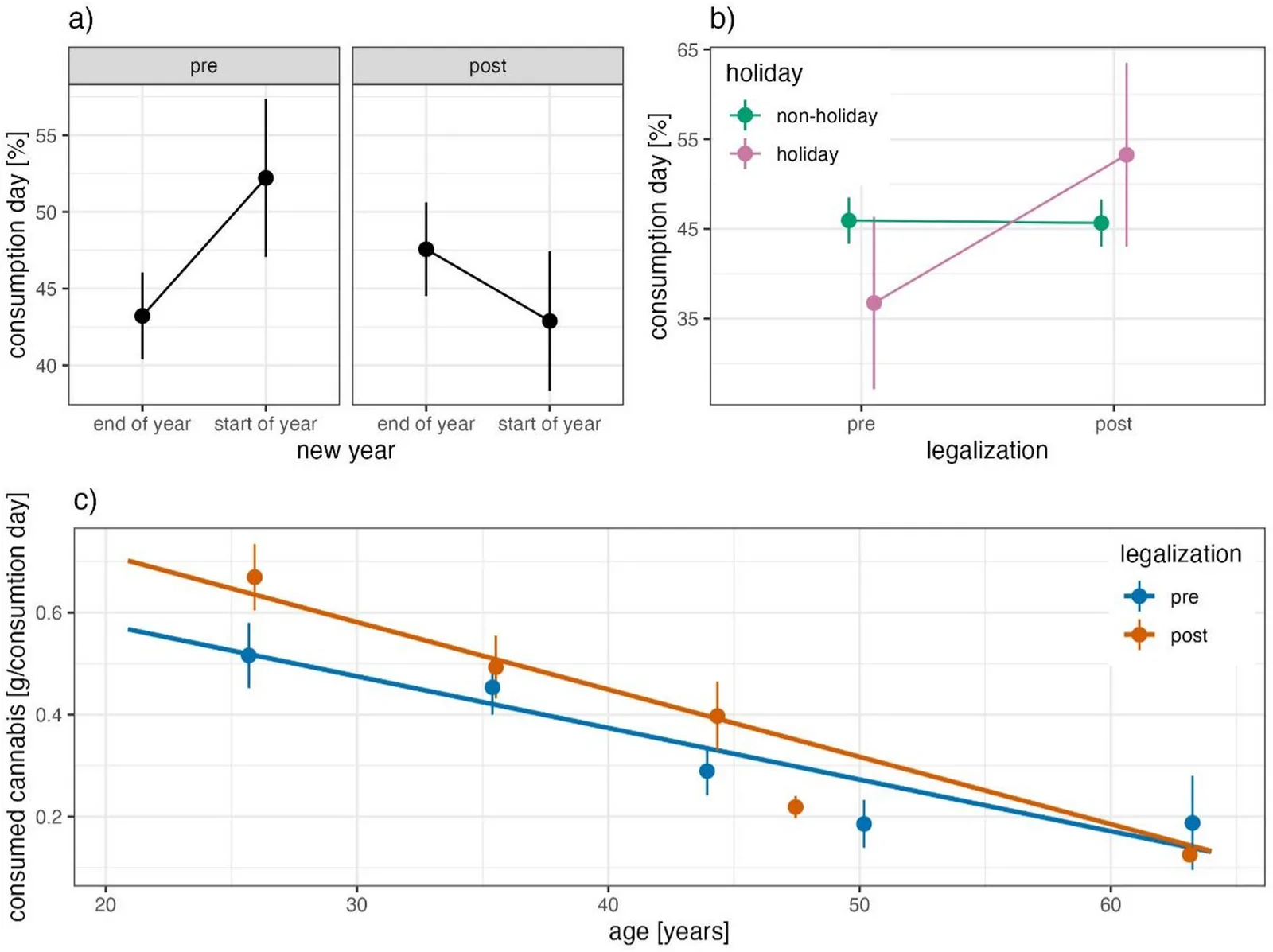
Cannabis legalization effects are shown for (**a**) end of year compared to start of year with a higher consumption frequency at start of year pre-legalization (β = 1.66, 95%CI = 1.16–2.37, z = 2.77, model 1). (**b**) consumption frequency on holidays with increased likelihood of consumption on holidays post-legalization (β = 3.91, 95%CI = 1.68–9.08, z = 3.17, model 1). (**c**) consumed cannabis amounts in g per consumption day across ages with a selective increase in consumption amounts of younger individuals (β = 0.01, 95%CI = 0.00. 0.02, z = 3.05).

Considering general consumption patterns centered at pre-legalization, participants were more likely to consume on weekend days compared to weekdays (β = 1.54, 95%CI = 1.12–2.12, z = 2.66, model 1), at the start of the year (β = 1.66, 95%CI = 1.16–2.37, z = 2.77, model 1), and were less likely to consume on holidays (β = 0.51, 95%CI = 0.29–0.92, z = −2.26, model 1). Age had a significant effect on the amount of cannabis consumed per day, with younger participants reporting higher consumption on days when they used cannabis (β = −0.03, 95%CI = −0.05 - −0.00, z = −2.33, model 2) (see Figs. 1 and 2, & Table 2).

**Table 2:** Model parameters for cannabis consumption days frequency (model 1) and consumed cannabis amounts on consumption days (model 2) as well as for location, setting and group frequencies on consumption days (model 3).

	Consumption days [model 1]				Consumed amounts [model 2]				Setting on consumption days [model 3]			
Fixed Effects												
Predictors	*β*	*95%CI*	*z*	*p*	*β*	*95%CI*	*z*	*p*	*β*	*95%CI*	*z*	*p*
(Grand mean)	0.65	0.27–1.55	−0.97	0.330	−1.36	−1.63 – −1.10	−10.25	**< 0.001**				
legalization [post]	1.60	0.99–2.58	1.92	0.054	0.14	−0.01–0.29	1.80	0.072				
weekday [weekend]	1.54	1.12–2.12	2.66	**0.008**	0.02	−0.07–0.11	0.39	0.695				
new year [start of year]	1.66	1.16–2.37	2.77	**0.006**	0.09	−0.01–0.19	1.73	0.083				
holiday [holiday]	0.51	0.29–0.92	−2.26	**0.024**	−0.02	−0.20–0.17	−0.17	0.866				
age	1.01	0.94–1.08	0.19	0.846	−0.03	−0.05 – −0.00	−2.33	**0.020**				
gender [non-woman]	1.13	0.21–6.03	0.15	0.884	0.14	−0.36–0.63	0.54	0.587				
legalization [post] × weekday [weekend]	1.19	0.76–1.87	0.77	0.444	0.06	−0.07–0.18	0.86	0.390				
legalization [post] × new year [start of year]	0.44	0.27–0.71	−3.33	**0.001**	−0.00	−0.14–0.13	−0.07	0.946				
legalization [post] × holiday [holiday]	3.91	1.68–9.08	3.17	**0.002**	0.18	−0.06–0.43	1.47	0.141				
legalization [post] × age	1.01	0.98–1.03	0.50	0.617	0.01	0.00–0.02	3.05	**0.002**				
legalization [post] × gender [non-woman]	0.89	0.54–1.46	−0.47	0.640	−0.15	−0.30–0.00	−1.95	0.052				
location [outdoor]					−0.09	−0.23–0.05	−1.23	0.219	0.47	0.30–0.75	−3.16	**0.002**
setting [public]					0.31	0.15–0.48	3.71	**< 0.001**	0.16	0.10–0.26	−7.39	**< 0.001**
group [group]					0.18	0.08–0.28	3.48	**0.001**	0.61	0.39–0.97	−2.10	**0.036**
legalization [post] × location [outdoor]					0.19	0.04–0.34	2.51	**0.012**	1.63	1.23–2.14	3.46	**0.001**
legalization [post] × setting [public]					−0.46	−0.66 – −0.25	−4.36	**< 0.001**	0.97	0.68–1.38	−0.18	0.854
legalization [post] × group [group]					−0.02	−0.16–0.11	−0.32	0.748	0.91	0.69–1.19	−0.71	0.479
Random Effects												
σ^2^	3.29				0.25				3.29			
τ_00_	6.97 _Subj_				0.53 _Subj_				1.84 _Subj_			
ICC	0.68				0.68				0.36			
N	49 _Subj_				44 _Subj_				44 _Subj_			
Observations	3020				1193				3579			
Marginal R^2^/Conditional R^2^	0.012/0.683				0.065/0.704				0.074/0.406			

Brackets in the fixed effects column refer to reference categories.

CI = Confidence Interval, ICC = Intraclass Correlation Coefficient, N = Number of random factor level.

Pre-to-post-legalization, an increase in consumption frequency at the start of the year was observed (β = 0.44, 95%CI = 0.27–0.71, z = −3.33, model 1), as well as an increased likelihood of consumption on holidays (β = 3.91, 95%CI = 1.68–9.08, z = 3.17, model 1). Additionally, the model a revealed significant of legalization with age for consumed amounts (β = 0.01, 95%CI = 0.00–0.02, z = 3.05, model 2) with younger individuals increasing their consumed cannabis amounts post regulation (see Fig. 2; Table 2).

Assessing the frequencies of outdoor, public and group consumption showed that participants were overall less likely to consume outdoor (β = 0.47, 95%CI = 0.30–0.75, z = −3.16, model 3), in public (β = 0.16, 95%CI = 0.10–0.26, z = −7.39, model 3), or in groups (β = 0.61, 95%CI = 0.39–0.97, z = −2.10, model 3). A legalization associated change was identified only for consumption location, with a significant increase in outdoor consumption from pre- to post-legalization (β = 1.63, 95%CI = 1.23–2.14, z = 3.46, model 3) (see Table 2).

**Fig. 3:**
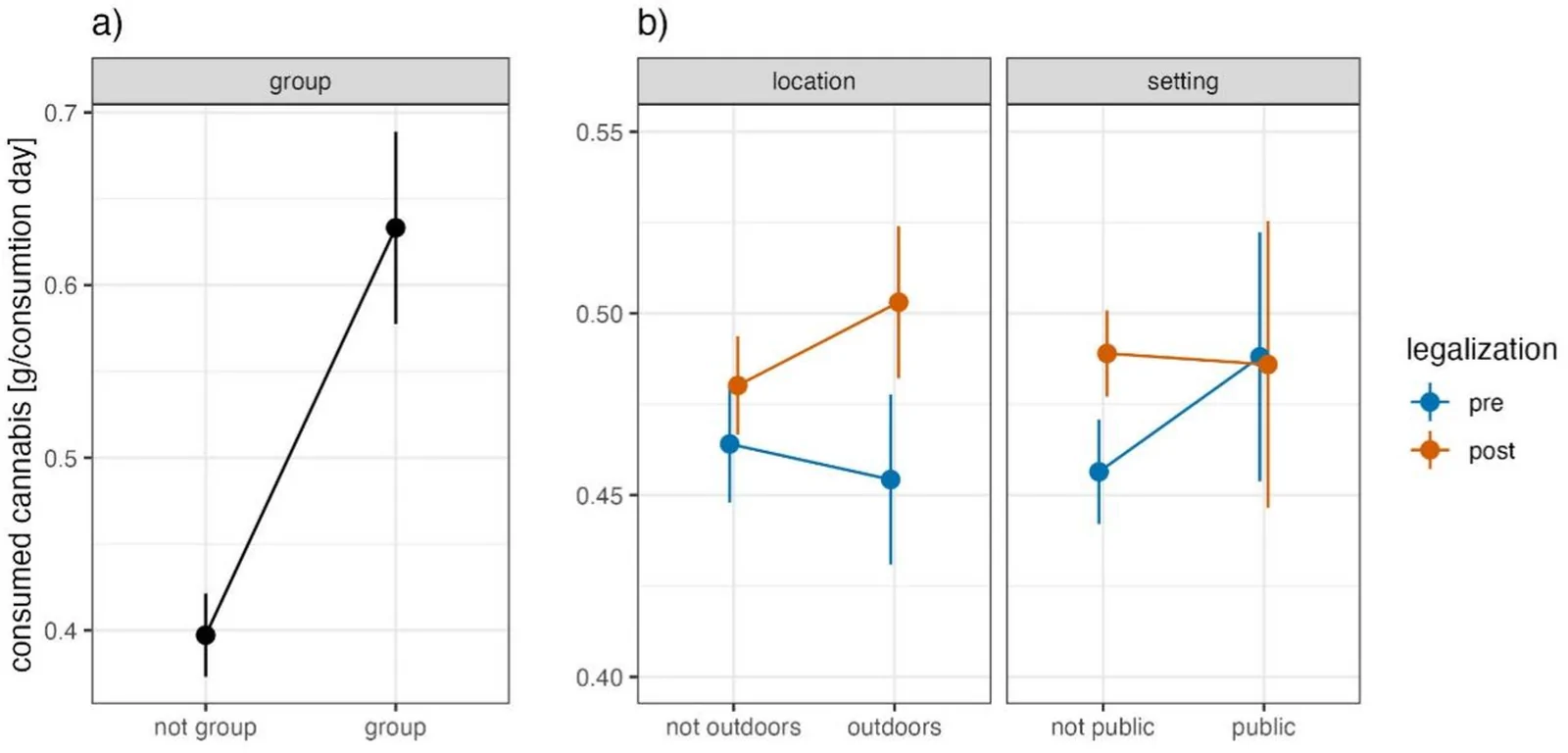
The effects of the setting on cannabis consumption pre- and post-legalization. The effects of the setting on cannabis consumption amounts in g per consumption day for (**a**) groups with higher consumption amounts in groups (β = 0.18, 95%CI = 0.08–0.28, z = 3.48, model 2). (**b**) public settings with a pre-to post-legalization decrease (β = −0.46, 95%CI = −0.66 - −0.25, z = −4.36) and an increase in outdoor settings (β = 0.19, 95%CI = 0.04–0.34, z = 2.51).

Results including context variables showed increases in consumption amounts when cannabis was consumed in groups (β = 0.18, 95%CI = 0.08–0.28, z = 3.48, model 2). Consumed amounts also increased for outdoor consumption from pre-to-post legalization (β = 0.19, 95%CI = 0.04–0.34, z = 2.51, model 2), while only non-public consumption amounts increased from pre-to-post legalization (β = −0.46, 95%CI = −0.66 - −0.25, z = −4.36, model 2) (see Fig. 3; Table 2).

Notably, implementing a social binary comparing women to men we found an interaction of gender with legalization for the consumed amounts of cannabis (β = −0.17, 95%CI = −0.32 - −0.01, z = −2.13) with women showing slightly larger increases in consumed amounts compared to men. However, this interaction was not present comparing women to non-women (see supplement Figure S3, Table S3 and model S3).

## Discussion

As an important addition to the debate on legalization of recreational cannabis use, this high-frequency EMA study provides real-life data on consumption behavior and the effects of Germany’s 2024 legal reform. Most notably, comparing two six-week observation periods from pre- to post-legalization, we observed an increase in consumption amounts of cannabis on consumption days selectively in young adults.

Pre-legalization, participants consumed cannabis on approximately 45.7% of observed days, with a mean of 0.475 g (approximately two mixed joints) per consumption day. These values are both lower compared to a similar sample from an EMA-cannabis study in the United States which may be reflected by a broader age range and a more detailed assessment of consumption modes (e.g., smoking, vaping, edibles) in this study^23^. Considering overall consumption patterns, cannabis consumption was more likely to occur on weekends which aligns with similar studies^18^^28,29^,–^30^.

Following the legalization, an increase in consumption amount on consumption days was selectively present in younger individuals. While an increase in consumption amounts was also observed in the majority of existing literature, we did not observe the similarly reported rise in use frequency^14–16^. A possible explanation lies in the sample composition: evidence from Canada and the US suggests that post-legalization increases in use frequency are primarily driven by new or returning users, whereas among pre-legalization users, frequency gains are smaller^14,16^. Furthermore, early implementation constraints can decouple consumption intensity from frequency as shown in Canada one month post-legalization, where limited access constrained opportunities to use more often while permitting heavier dosing^16^ Germany’s phased approach and initially limited legal availability plausibly mirror these conditions.

The increase of cannabis consumption amount per use day pre- to post-legalization selectively occurred in younger individuals which has not been shown in comparable studies before^14,15^–^16^. This is potentially clinically relevant given dose-dependent risks, including the development or worsening of cannabis usage disorder (CUD) and mental health symptoms such as the incidence of psychotic disorder^3–6,31,32^.. In our study, younger adults (20.9–24.4 years) consumed twice as much cannabis as older adults (38.7–64 years) pre- to post-legalization. This is approximately half a joint more per day in younger adults and about a third of a joint more in older adults (see Supplement Figure S2 and Table S2). Prior research indicates that younger individuals tend to consume cannabis more frequently and intensely, suggesting that their consumption may be more susceptible to social changes^33^. A key challenge in contextualizing these findings lies in the multitude of political, social, and cultural factors shaping outcomes within specific settings. For Germany, directly comparable data are currently not yet available, underlining the relevance of this study.

Furthermore, analyses revealed a lower likelihood of cannabis consumption pre-legalization on holidays, which is opposite to previously published patterns in alcohol consumption^18^. Interestingly, this cannabis consumption pattern adjusted pre- to post-legalization, indicating that the change of legalization might have increased the overall acceptability of cannabis consumption, which in turn increased the likelihood of consumption during holidays with more social settings such as Christmas or New Year’s Eve.

As previous studies revealed that cannabis consumption increased around the date of the legalization, it is possible that the increase in consumption frequency at the start of the year pre-legalization can be interpreted as a so-called anticipation effect^16^. Specifically, consumption may have increased through the immediacy, media presence, and novelty of the legalization procedures.

Finally, we examined setting-specific effects of legalization, an understudied aspect in cannabis research. Analyses indicated that while the frequency of group consumption has declined, the quantity consumed per social session increased, mirroring trends in alcohol use^23,25^. Despite this lower frequency of occurrence, the intensity of outdoor consumption has significantly escalated, with participants reporting increased cannabis amounts when consuming in outdoor environments. These findings are consistent with studies in the US, which may reflect early destigmatization and a diminished perception of legal risk^17^.

The hypothesis of increased public consumption following legalization, through removal of legal penalties lowering barriers to open use, was not supported. Instead, public consumption remained unaffected by legalization. This may reflect ongoing regulatory uncertainty and persistent social norms that may have limited consumption in clearly public areas. Further, specifically in Germany, public consumption was limited legally (e.g. in sight of schools, children’s and youth facilities or publicly accessible sports facilities). By capturing the immediate post-legalization period, these findings contribute important early insights to inform prevention, treatment, and regulation. Further changes in use patterns may emerge over time and should be examined within their evolving sociopolitical context to assess more stable trajectories in the future.

The generalizability of our findings may be limited, given that recruitment occurred primarily within clinical and university-affiliated settings. The resulting sample likely included participants who already perceived the legal risks of cannabis use as relatively low and who were in more stable life circumstances. Importantly, our study focused exclusively on participants who already used cannabis prior to legalization. In addition, the sample size, while methodologically adequate for EMA designs, further constrains the generalizability of results. A further limitation concerns the measurement of cannabis quantities: self-reported cannabis quantities in grams is limited due to the absence of standardized dosing guidelines and legal quality controls. Furthermore, as legalization may reduce perceived stigma or fear of legal consequences, participants could have become more willing to report their use accurately, potentially contributing to observed changes over time. Finally, although EMA enhances ecological validity, the method is susceptible to reactivity, where repeated self-monitoring may itself influence use patterns.

## Conclusion

This six-week pre/post legalization EMA-study shows that the cannabis legalization in Germany was followed by an increase in cannabis consumption amounts on consumption days selectively in young adults, whereas the frequency of consumption days was not altered.

A potential anticipation effect of the legalization may have led to higher consumption frequencies at the start of the year pre-legalization with a following decrease in cannabis consumption days at the same period post-legalization. Following the legalization, a higher likelihood for consumption on holidays, along with shifts in context, including greater use in outdoor and social but not in public settings was observed.

Taken together, these changes suggest that legalization can alter not only how often cannabis is used, but also how much, where, and with whom. Clinically, this underscores the importance of assessing consumption intensity and social-contextual factors when evaluating cannabis-related risks and tailoring interventions in the evolving legal landscape. Given the early stage of implementation, further studies are needed to capture longer-term developments particularly in vulnerable subgroups such as younger individuals and assess whether observed patterns stabilize, intensify, or shift over time.

## Methods

### Participants and study design

Participants were recruited from the cohort of the Collaborative Research Centre TRR265^34^. Additionally, we recruited participants via flyers/advertisement distributed in psychiatric clinics, universities, and social venues such as bars across Berlin. Inclusion criteria were current non-medical cannabis use (at least once per month), age of 18 years or older, no night shift work (as momentary elements of EMA were structured after the daily routine of a day job) and fluent German language skills. Exclusion criteria included pregnancy, signs of severe adverse health conditions (e.g., chronic disease, disease of the central nervous system, mania, bipolar disorder, schizophrenia, severe depressive episode), current substance use disorder other than alcohol usage disorder (AUD), tobacco use disorder (TUD) or cannabis use disorder (CUD), risk for suicide, or severe withdrawal symptoms. Data collection occurred in two periods: from November 26, 2023, to February 14, 2024 (pre-legalization), and from November 26, 2024, to February 24, 2025 (post-legalization) assessing daily cannabis consumption over 6 weeks. This study was performed in accordance with relevant guidelines and regulations, including the Declaration of Helsinki and was approved by the Ethics Committee at Charité – Universitätsmedizin Berlin (reference no. EA1/77/2022). All participants provided written informed consent prior to participation.

### Ecological momentary assessment

#### Cannabis consumption

Cannabis use was assessed using the smartphone application “movisensXS” (movisens GmbH, Karlsruhe, Germany), which complies with both the General Data Protection Regulation (GDPR) of the European Union and the Berlin Data Protection Act (Berliner Datenschutzgesetz – BlnDSG). The EMA consisted of repeated sampling of participants’ real-time behaviors and experiences such as daily inquiring of yesterdays consumed cannabis amounts based on the utilized method (i.e., mixed (tobacco & cannabis) or pure joint, edibles, special smoking methods). Consumed amounts were assessed in gram with estimation helpers for mixed joints (~ 0.25 g per joint), pure joints (~ 0.5 g per joint), and edibles (~ 0.1–0.4 g per edible). From these assessments we derived a binary measure coding consumption day to estimate the frequency of consumption days, as well as the amounts of consumed cannabis in gram per consumption day. Prompts were sent out every day at 12:00.

#### Cannabis consumption setting

Additionally, contextual information for cannabis consumption days was collected every other day for the previous two days including the consumption location (i.e., indoor and/or outdoor), setting (i.e., public and/or private), and group (i.e., alone, in pairs, small group (2–4), and/or large group (+ 4)). Due to low numbers of days with multiple responses for each item, true binaries were derived (i.e., location was coded to compare days with any outdoor consumption to days without any outdoor consumption, setting as days with and without public consumption and groups as days with and without group consumption).

#### Additional variables

Age was calculated for the onset of the pre-legalization assessment and gender was assessed using the options woman, man and non-binary. Due to two participants reporting a non-binary gender, gender was coded as a binary variable for this analysis (women vs. non-women). Concerning temporal variation, a binary weekday variable was generated allowing for comparison of days preceding the weekend (Friday and Saturday) vs. other weekdays. Similarly, a binary holiday variable was generated for days followed by an official bank holiday, and a new year’s variable was generated as the assessments encompassed the transition for one year into another. Here consumption at the end of the year was compared to consumption at the start of the year. Finally, a legalization variable was implemented comparing consumption before versus after legalization on April 1, 2024.

### Statistical analysis

We employed linear mixed model approaches for the following three models: In **model 1** we assessed effects of legalization on frequency of cannabis consumption days using a generalized linear mixed model (GLMM) approach, in **model 2** we assessed the effects of legalization on the consumed amounts on consumption days utilizing a linear mixed models (LMM) approach, and in **model 3** we assessed the effects of legalization on the setting on consumption days using a GLMM approach. Box-Cox analyses suggested log transformation of consumption amounts to achieve more normally distributed data. Treatment contrasts were used for the legalization, centering the pre-legalization period. For all other binary variables (i.e., weekday, holiday, gender, location, setting, and group) sequential differences contrasts were applied to avoid bias in the estimation of the model’s grand mean. Aside from legalization, model 1 and 2 included age, gender, weekday, holiday and new year, as well as their interactions with legalization. Model 2 and 3 further included consumption location, setting, and group, as well as their interactions with legalization. |z-values| ≥ 2.0 were interpreted as statistically significant. All models were structured as two-level models accounting for daily information (level 1) and individual differences on a subject level (level 2) accounting for individual differences. All analyses were conducted using R (via RStudio, Version 2025.05.1 + 513). Utilized packages used for data management, visualization, and model diagnostics included lme4, tidyverse^35^, summarytools^36^, jPlot^37^, PerformanceAnalytics^38^, and gt^39^. Rendered scripts are made publicly available (https://osf.io/3qcrk/overview?view_only=fe7f675770ef4b41a9e8ab3334e32b51).

## Supplementary Information

Below is the link to the electronic supplementary material.

Supplementary Material 1

## Data Availability

The datasets generated and analyzed during the current study are not publicly available to protect the privacy of the participants but are available from the corresponding author on reasonable request. The scripts and code used for data analysis is publicly available under: https://osf.io/3qcrk/overview?view_only=fe7f675770ef4b41a9e8ab3334e32b51.

## References

[1] World Drug Report. *United Nations: Office on Drugs and Crime* (2021). https://www.unodc.org/unodc/en/data-and-analysis/wdr2021.html

[2] Rauschert, C. et al. Kurzbericht Epidemiologischer Suchtsurvey 2021. (2023).

[3] Marconi, A., Di Forti, M., Lewis, C. M., Murray, R. M. & Vassos, E. Meta-analysis of the association between the level of cannabis use and risk of psychosis. *Schizophr. Bull.* **42**, 1262–1269 (2016). 26884547 10.1093/schbul/sbw003 PMC4988731

[4] Lev-Ran, S. et al. The association between cannabis use and depression: a systematic review and meta-analysis of longitudinal studies. *Psychol. Med.* **44**, 797–810 (2014). 23795762 10.1017/S0033291713001438

[5] Xue, S., Husain, M. I., Zhao, H. & Ravindran, A. V. Cannabis Use and Prospective Long-Term Association with Anxiety: A Systematic Review and Meta-Analysis of Longitudinal Studies: Usage du cannabis et association prospective à long terme avec l’anxiété: une revue systématique et une méta-analyse d’études longitudinales. *Can. J. Psychiatry* **66**, 126–138 (2021). 32909828 10.1177/0706743720952251 PMC7918873

[6] Moore, T. H. M. et al. Cannabis use and risk of psychotic or affective mental health outcomes: a systematic review. *Lancet Lond. Engl.* **370**, 319–328 (2007). 10.1016/S0140-6736(07)61162-3 17662880

[7] Potter, G. R. & Wells, H. More harm than good? Cannabis, harm and the misuse of drugs act. *Drugs and Alcohol Today* **21**, 277–288 (2021).

[8] Debating the legalisation of recreational cannabis. *Lancet Reg. Health - Eur.* **10**, 100269 (2021). 34826314 10.1016/j.lanepe.2021.100269 PMC8589712

[9] Farrelly, K. N. et al. The Impact of Recreational Cannabis Legalization on Cannabis Use and Associated Outcomes: A Systematic Review. *Subst. Abuse Res. Treat.* **17**, 11782218231172054 (2023). 10.1177/11782218231172054 PMC10176789 37187466

[10] Bundesgesetzblatt Teil I - Gesetz zum kontrollierten Umgang mit Cannabis und zur Änderung weiterer Vorschriften - Bundesgesetzblatt. https://www.recht.bund.de/bgbl/1/2024/109/VO.html?nn=55638

[11] Fragen und Antworten zum Cannabisgesetz. *BMG* https://www.bundesgesundheitsministerium.de/themen/cannabis/faq-cannabisgesetz.html

[12] Poulton, A., Pan, J., Bruns, L. R., Sinnott, R. O. & Hester, R. Assessment of alcohol intake: Retrospective measures versus a smartphone application. *Addict. Behav.* **83**, 35–41 (2018). 29128148 10.1016/j.addbeh.2017.11.003

[13] Trull, T. J. & Ebner-Priemer, U. W. Using experience sampling methods/ecological momentary assessment (ESM/EMA) in clinical assessment and clinical research: Introduction to the special section. *Psychol. Assess.* **21**, 457–462 (2009). 19947780 10.1037/a0017653 PMC4255457

[14] Gunadi, C., Zhu, B. & Shi, Y. Recreational cannabis legalization and transitions in cannabis use: findings from a nationally representative longitudinal cohort in the United States. *Addict. Abingdon Engl.* **117**, 2651–2659 (2022). 10.1111/add.15895 PMC11147131 35618659

[15] Gali, K., Winter, S. J., Ahuja, N. J., Frank, E. & Prochaska, J. J. Changes in cannabis use, exposure, and health perceptions following legalization of adult recreational cannabis use in California: A prospective observational study. *Subst. Abuse Treat. Prev. Policy* **16**, 16 (2021). 33579324 10.1186/s13011-021-00352-3 PMC7881543

[16] Turna, J. et al. Cannabis use and misuse in the year following recreational cannabis legalization in Canada: A longitudinal observational cohort study of community adults in Ontario. *Drug Alcohol Depend.* **225**, 108781 (2021). 34098383 10.1016/j.drugalcdep.2021.108781

[17] Carliner, H., Brown, Q. L., Sarvet, A. L. & Hasin, D. S. Cannabis use, attitudes, and legal status in the U.S.: A review. *Prev. Med.* **104**, 13–23 (2017). 28705601 10.1016/j.ypmed.2017.07.008 PMC6348863

[18] Deeken, F. et al. Patterns of alcohol consumption among individuals with alcohol use disorder during the COVID-19 pandemic and lockdowns in Germany. *JAMA Netw. Open* **5**, e2224641 (2022). 10.1001/jamanetworkopen.2022.24641 PMC9344361 35913741

[19] de Visser, R. O., Robinson, E. & Bond, R. Voluntary temporary abstinence from alcohol during ‘Dry January’ and subsequent alcohol use. *Health Psychol.* **35**, 281–289 (2016). 26690637 10.1037/hea0000297

[20] Hall, W. What has research over the past two decades revealed about the adverse health effects of recreational cannabis use? *Addiction* **110**, 19–35 (2015). 25287883 10.1111/add.12703

[21] Malone, D. T., Hill, M. N. & Rubino, T. Adolescent cannabis use and psychosis: epidemiology and neurodevelopmental models. *Br. J. Pharmacol.* **160**, 511–522 (2010). 20590561 10.1111/j.1476-5381.2010.00721.x PMC2931552

[22] Jacobus, J., Bava, S., Cohen-Zion, M., Mahmood, O. & Tapert, S. F. Functional consequences of marijuana use in adolescents. *Pharmacol. Biochem. Behav.* **92**, 559–565 (2009). 19348837 10.1016/j.pbb.2009.04.001 PMC2697065

[23] Ching-Yun, Y. et al. Predicting quantity of cannabis smoked in daily life: An exploratory study using machine learning. *Drug Alcohol Depend.* **252**, 110964 (2023). 37748423 10.1016/j.drugalcdep.2023.110964 PMC10615868

[24] Phillips, K. T., Phillips, M. M., Lalonde, T. L. & Prince, M. A. Does social context matter? An ecological momentary assessment study of marijuana use among college students. *Addict. Behav.* **83**, 154–159 (2018). 29329753 10.1016/j.addbeh.2018.01.004

[25] Fischer, A. M., King, A. C., Cao, D. & Fridberg, D. J. Drinking context, alcohol use, and subjective responses during binge-drinking episodes measured by high-resolution ecological momentary assessment (HR-EMA). *Psychol. Addict. Behav.* **37**, 258–266 (2023). 36326673 10.1037/adb0000887 PMC9991931

[26] Corbin, W. R., Scott, C., Boyd, S. J., Menary, K. R. & Enders, C. K. Contextual influences on subjective and behavioral responses to alcohol. *Exp. Clin. Psychopharmacol.* **23**, 59–70 (2015). 25643028 10.1037/a0038760

[27] Monk, R. L., Qureshi, A. & Heim, D. An examination of the extent to which mood and context are associated with real-time alcohol consumption. *Drug Alcohol Depend.* **208**, 107880 (2020). 32004997 10.1016/j.drugalcdep.2020.107880

[28] Buckner, J. D., Henslee, A. M. & Jeffries, E. R. Event-specific cannabis use and use-related impairment: The relationship to campus traditions. *J. Stud. Alcohol Drugs* **76**, 190–194 (2015). 25785793 10.15288/jsad.2015.76.190 PMC5374473

[29] Buckner, J. D., Walukevich, K. A. & Lewis, E. M. Cannabis use motives on weekends versus weekdays: Direct and indirect relations with cannabis use and related problems. *Addict. Behav.* **88**, 56–60 (2019). 30142485 10.1016/j.addbeh.2018.08.012

[30] Wenzel, J. G. et al. One-year ecological momentary assessment of alcohol use, mood, and stress among individuals with alcohol use disorder during SARS-CoV-2 pandemics: a gender-specific reflection. *Eur. Arch. Psychiatry Clin. Neurosci.* **275**, 451–461 (2025). 39560734 10.1007/s00406-024-01930-9 PMC11910400

[31] Callaghan, R. C., Sanches, M. & Kish, S. J. Quantity and frequency of cannabis use in relation to cannabis-use disorder and cannabis-related problems. *Drug Alcohol Depend.* **217**, 108271 (2020). 32977043 10.1016/j.drugalcdep.2020.108271

[32] Di Forti, M. et al. The contribution of cannabis use to variation in the incidence of psychotic disorder across Europe (EU-GEI): a multicentre case-control study. *Lancet Psychiatry*. **6**, 427–436 (2019). 30902669 10.1016/S2215-0366(19)30048-3 PMC7646282

[33] Doggett, A. et al. Cannabis Use Frequency and Cannabis-Related Consequences in High-Risk Young Adults Across Cannabis Legalization. *JAMA Netw. Open.* **6**, e2336035 (2023). 37755827 10.1001/jamanetworkopen.2023.36035 PMC10534274

[34] Spanagel, R. et al. The ReCoDe addiction research consortium: Losing and regaining control over drug intake-Findings and future perspectives. *Addict. Biol.* **29**, e13419 (2024). 38949209 10.1111/adb.13419 PMC11215792

[35] Wickham, H. et al. Welcome to the tidyverse. *J. Open Source Softw.* **4**, 1686 (2019).

[36] Comtois, D. & summarytools Tools to Quickly and Neatly Summarize Data. (2025).

[37] Lüdecke, D. & sjPlot Data Visualization for Statistics in Social Science. (2025).

[38] Peterson, B. G., Carl, P. & PerformanceAnalytics Econometric Tools for Performance and Risk Analysis. 2.0.8 (2007). 10.32614/CRAN.package.PerformanceAnalytics

[39] Iannone, R. et al. gt: Easily Create Presentation-Ready Display Table 1.0.0 (2020). 10.32614/CRAN.package.gt

[40] Babor, T. F., Higgins-Biddle, J. C., Saunders, J. B. & Monteiro, M. G. *AUDIT The Alcohol Use Disorders Identification Test: Guidelines for Use in Primary Care* (World Health Organization, Department of Mental Health and Substance Dependence, 2001).

[41] Adamson, S. J. & Sellman, J. D. A prototype screening instrument for cannabis use disorder: The Cannabis Use Disorders Identification Test (CUDIT) in an alcohol-dependent clinical sample. *Drug Alcohol Rev.* **22**, 309–315 (2003). 15385225 10.1080/0959523031000154454

