# Supplementary material for: Ecological momentary insights into cannabis use and its contexts: a pre-post legalization study in Germany
Source: Sci Rep. 2026 Sep 5;16:27818. doi: 10.1038/s41598-026-70148-7 (PMC13546282; doi:10.1038/s41598-026-70148-7)
Supplement: Supplementary file 1 [file 41598_2026_70148_MOESM1_ESM.docx]

Supplement

Rendered scripts are made publicly available: osf.io/3qcrk

Supplement Figure S1. Flowchart of the recruitment process.

Supplement Figure S2. Differences of consumed Cannabis [g/consumption day] pre-/to post-legalization in four equal sized age groups (20.9-24.4; 24.4-31.2; 31.2-38.7; 38.7-64 years).

Supplement Table S2.

| age_group | N | pre | post | diff |
| --- | --- | --- | --- | --- |
| [20.9,24.4] | 11 | 0.70±0.16 | 0.86±0.14 | 0.16 |
| [24.4,31.2] | 11 | 0.42±0.04 | 0.52±0.05 | 0.10 |
| [31.2,38.7] | 11 | 0.46±0.06 | 0.51±0.07 | 0.05 |
| [38.7,64] | 11 | 0.26±0.04 | 0.34±0.05 | 0.08 |

|  | **Consumption days**  **[model 1]** | | | | **Consumed amounts**  **[model 2]** | | | |
| --- | --- | --- | --- | --- | --- | --- | --- | --- |
| *Predictors* | *β* | *95%CI* | *z* | *p* | *β* | *95%CI* | *z* | *p* |
| *(Grand mean)* | 0.60 | 0.26 – 1.42 | -1.16 | 0.246 | -1.37 | -1.62 – -1.11 | -10.60 | <0.001 |
| *legalization [post]* | 1.61 | 1.00 – 2.60 | 1.96 | 0.049 | 0.11 | -0.04 – 0.27 | 1.44 | 0.149 |
| *weekday [weekend]* | 1.53 | 1.11 – 2.10 | 2.62 | 0.009 | 0.01 | -0.09 – 0.10 | 0.11 | 0.910 |
| *new year [start of year]* | 1.65 | 1.15 – 2.36 | 2.74 | 0.006 | 0.07 | -0.03 – 0.17 | 1.35 | 0.178 |
| *holiday [holiday]* | 0.51 | 0.28 – 0.91 | -2.29 | 0.022 | 0.01 | -0.19 – 0.21 | 0.07 | 0.945 |
| *age* | 1.01 | 0.94 – 1.08 | 0.16 | 0.875 | -0.03 | -0.05 – -0.01 | -2.49 | 0.013 |
| *gender [man]* | 1.00 | 0.19 – 5.17 | -0.00 | 0.998 | 0.08 | -0.39 – 0.55 | 0.34 | 0.732 |
| *legalization [post] × weekday [weekend]* | 1.20 | 0.77 – 1.88 | 0.80 | 0.426 | 0.07 | -0.06 – 0.20 | 1.02 | 0.310 |
| *legalization [post] × new year [start of year]* | 0.44 | 0.27 – 0.72 | -3.29 | 0.001 | 0.02 | -0.12 – 0.15 | 0.23 | 0.819 |
| *legalization [post] × holiday [holiday]* | 3.97 | 1.70 – 9.24 | 3.20 | 0.001 | 0.17 | -0.09 – 0.42 | 1.28 | 0.200 |
| *legalization [post] × age* | 1.01 | 0.98 – 1.03 | 0.50 | 0.620 | 0.01 | 0.00 – 0.02 | 2.81 | 0.005 |
| *legalization [post] × gender [man]* | 0.89 | 0.54 – 1.46 | -0.48 | 0.633 | -0.17 | -0.32 – -0.01 | -2.13 | 0.034 |
| *location [outdoor]* |  |  |  |  | -0.11 | -0.26 – 0.04 | -1.41 | 0.159 |
| *setting [public]* |  |  |  |  | 0.36 | 0.18 – 0.54 | 4.00 | <0.001 |
| *group [group]* |  |  |  |  | 0.20 | 0.10 – 0.31 | 3.73 | <0.001 |
| *legalization [post] × location [outdoor]* |  |  |  |  | 0.21 | 0.05 – 0.36 | 2.59 | 0.010 |
| *legalization [post] × setting [public]* |  |  |  |  | -0.50 | -0.71 – -0.28 | -4.52 | <0.001 |
| *legalization [post] × group [group]* |  |  |  |  | -0.06 | -0.20 – 0.08 | -0.84 | 0.399 |
| *Random Effects* | | | | | | | | |
| *σ^2^* | 3.29 | | | | 0.25 | | | |
| *τ_00_* | 6.67 _Subj_ | | | | 0.47 _Subj_ | | | |
| *ICC* | 0.67 | | | | 0.65 | | | |
| *N* | 48 _Subj_ | | | | 43 _Subj_ | | | |
| *Observations* | 2963 | | | | 1140 | | | |
| *Marginal R^2^ / Conditional R^2^* | 0.012 / 0.673 | | | | 0.080 / 0.678 | | | |
| *CI = Confidence Interval, ICC = Intraclass Correlation Coefficient, N = Number of random factor level* | | | | | | | | |

Notes. Differences of consumed Cannabis [g/consumption day] pre-/to post-legalization in different age groups. Participants between 38.7-64 years consumed 0.08 g (approximately a third joint more calculated for mixed Joints with ~0.25g/ Cannabis) more per day, whereas participants from 20.9-24.4 consumed 0.16g more per day (more than half a Joint more

Supplement Model S2.

Supplement Figure S3: a) depicts consumed cannabis on consumption days mean ± CI comparing woman with non-woman pre to post legalization; b) depicts consumed cannabis on consumption days mean ± CI comparing woman with man pre to post legalization; Model predictors were used for both figures.

Supplement Table S3.

| gender | pre | post |
| --- | --- | --- |
| **social binary** | | |
| woman | 0.45±0.02 | 0.48±0.02 |
| man | 0.42±0.02 | 0.44±0.01 |
| **true binary** | | |
| woman | 0.47±0.02 | 0.51±0.02 |
| non-woman | 0.46±0.02 | 0.48±0.01 |
